# Editorial: Community series in immunoregulation at mucosal surfaces volume II

**DOI:** 10.3389/fimmu.2023.1294936

**Published:** 2023-10-09

**Authors:** Javier Leceta, Christoph S. N. Klose, Stefan Jordan

**Affiliations:** ^1^ Department of Cell Biology, Faculty of Biology, Complutense University of Madrid, Madrid, Spain; ^2^ Department of Microbiology, Infectious Diseases and Immunology, Charité – Universitätsmedizin Berlin, corporate member of Freie Universität Berlin and Humboldt-Universität zu Berlin, Berlin, Germany

**Keywords:** mucosal immunity, innate immune system, immunoregulation, immune mechanism, animal model

The epithelia lining the surfaces in contact with the external environment are represented by the intestinal, respiratory, urinary, and skin epithelia. These locations represent, in surface area and volume, the largest component of the individual’s immune system. They integrate both pathogens and microorganism symbionts with the defense system. In these locations, there are also different cell types, such as epithelial cells, lymphoid cells, and non-lymphoid cells, that carry out the defense mechanisms of both the innate and acquired immune response. The immune response in the mucous membranes also regulates the peripheral immune response. This Research Topic mainly provides articles studying the mechanisms of the innate immune response or its impact on the peripheral immune response.

The article by French et al. compares the course of the response to infection with Toxoplasma gondii according to the routes of infection, focusing on brain inflammation and changes in the microbiota. T. gondii is a parasite that infects between 30 and 70% of the human population. In experimental models, oral (p.o.) or intraperitoneal (i.p.) infection has been used to study the characteristics of the mucosal immune response or the peripheral immune response, respectively. The article describes how i.p.-infected mice develop a more severe course of infection, characterized by a large increase in pro-inflammatory cytokines, particularly in the CNS, associated with a reduction in the expression of genes involved in the transmission of neuronal signals. On the other hand, p.o. infection is associated with an increased intestinal inflammatory response. In both cases, dysbiosis occurs, which is more accentuated by p.o. immunization. These data highlight the relationship between peripheral inflammation and CNS homeostasis.

The article by Goswamy et al. analyzes the mechanism of regulation by TFEB transcription factors from the family of Microphthalmia/TFE (MiT) transcription factors (HLH30 in Caenorhabditis elegans), which regulate inflammation and innate immunity in both invertebrates and vertebrates. In C. elegans, oral infection with Staphylococcus aureus causes a 7-fold reduction in its life span. HLH-30 directly or indirectly induces the expression of about 600 genes during S. aureus infection. However, 90% of these genes do not have regulatory sequences recognized by HLH-30, suggesting the possibility that HLH-30 exerts its action through other transcription factors induced by HLH-30. Indeed, HLH-30 induces the expression of 17 transcription factors during S. aureus infection. The article identifies that one of these factors, nuclear factor NHR42, is a negative regulator of resistance to infection in the intestinal epithelium, as deduced from an increased survival of mutants of this gene to S. aureus infection. NHR42 is also expressed in uninfected worms in an independent HLH-30 manner but increases its activity after infection. NHR-42 mutants show increased expression of genes related to various innate immunity and host defense categories. The nhr42 mutants also over-represent genes related to lipid metabolism.

The article by Patoine et al. studies the role of the CD200/CD200R system, an important anti-inflammatory pathway, in a model of lung inflammation by LPS, where these molecules have high expression rates, especially in macrophages. The cells of the innate immune system are key to the defense mechanisms of the mucous membranes. They are responsible for triggering inflammatory responses but also in resolving inflammation. An imbalance between initiation and resolution is the cause of important pathologies. KO rats for CD200 develop greater pulmonary edema of higher numbers of neutrophils and higher levels of TNF, IL-6, and CCL2. These effects are reversed by the administration of CD200Fc, so the authors conclude that the administration of CD200 agonists may have a beneficial effect on inflammatory lung diseases.

Walker et al.‘s article discusses the role of the immune response beyond conventional CD4 cells in protecting against the development of tuberculosis. It characterizes the ex vivo response of reactive innate T cells against Bacillus Calmette–Guérin (BCG) lipids, both in peripheral blood and bronchioalveolar lavage. It uses macaques inoculated with BCG by aerosols before and after infection with SIV as a model. It highlights the importance of unconventional T cells reactive to mycobacterial lipids after exposure to BCG, which, however, are lower in macaques infected with SIV. Even in this case, the cytolytic potential of these cells is maintained. The importance of the study lies in its possible application in cases of HIV infection. Although BCG vaccination protects against tuberculosis in children, its efficacy in adults is limited, and AIDS patients have a 20-fold higher risk of tuberculosis and a 4-fold higher mortality.

Chen and Wang‘s review discusses the mechanisms underlying olfactory dysfunction (OD) frequently associated with COVID-19 infection. The olfactory epithelium is a specialized epithelium of the nasal cavity formed by several cell types. Olfactory neurons are bipolar neurons with specialized apical cilia in which odor receptors are located and basal axons that meet on the olfactory nerve. They are surrounded by non-neuronal supporting cells of two types. The sustentacular cells (SUS) represent the physical support of the olfactory epithelium and have metabolic functions. They represent the main entry point of COVID-19 by expressing the ACE2 receptor for the entry of the virus. COVID-19 infection reduces the number of SUS and increases immune cells in the olfactory epithelium. The review also describes the involvement of SUS and the inflammatory response of the nasal epithelium in the DO process and discusses the effect of various inflammatory mediators on olfactory neuron response, SUS, and neuroepithelial regeneration.

Innate lymphoid cells (ILCs) are preferably located in the mucous membranes and are the first defense barrier against pathogens, also intervening in repair processes and development of the immune response. In their article, Castillo-Gonzales et al. review the role of ILC3s in carcinogenic processes, particularly in mucous membranes. Both protumor and antitumor effects of ILC3s have been described. The production of IL-22 by these cells associated with intestinal inflammatory processes has been linked to colorectal cancer (CRC) since IL-22 promotes the proliferation of intestinal epithelial stem cells. Moreover, IL-22 initiates a DNA damage response in intestinal epithelial stem cells that can prevent CRC initiation. The authors extend their review to other types of cancer (skin, breast, pancreatic, and lung cancers). They suggest that a better knowledge of the cytokine environment and its temporal appearance in tumors and a better characterization of ILC3s subtypes would help to solve the controversy of their involvement in tumorigenic processes.

## Author contributions

JL: Writing – original draft. CK: Visualization, Writing – review & editing. SJ: Writing – review & editing.

